# The Influence of Infant Schema Cues on Donation Intention in Charity Promotion

**DOI:** 10.3389/fpsyg.2022.869458

**Published:** 2022-07-12

**Authors:** Chen Yang, Mengying Zhao, Chunya Xie, Jingyi Li

**Affiliations:** ^1^Renmin Business School, Renmin University of China, Beijing, China; ^2^College of Economics and Management, Zhejiang A&F University, Hangzhou, China

**Keywords:** infant schema, empathy, donation intention, charity promotion, gender

## Abstract

This research performed four experiments to investigate the influence of infant schema cues on charitable donation intention and examine the moderating effect of gender. The results indicate that: (1) individuals stimulated by infant schema (vs. adult schema) cues had a higher willingness to donate when facing charity promotion; (2) the main effect was not due to the perceived cuteness of character in posters; (3) empathy played an entirely mediating role in the relationship between infant schema cues and donation intention; (4) gender moderated the influence of infant schema on donation intention: infant schema cues are effective for improving females’ donation intention, but ineffective for males. These findings contributed to the literature on infant schema and provide practical significance for introducing infant schema in charity promotion.

## Introduction

Charity plays an essential role in maintaining social stability and enhancing people’s sense of responsibility. A charitable donation is the third distribution of national income, which plays an important role in narrowing the income distribution gap and promoting social equity. According to the 2020 China Charitable Donation Report released by the Chinese Academy of Social Sciences, the Chinese mainland received 208.61 billion yuan in donations in 2020, accounting for 0.21% of its GDP. Individual donations in mainland China totaled 52.41 billion yuan, up 31.5 percent year on year, and it surpassed the amount donated after the 2008 Wenchuan earthquake to become the first. Individual donations have been at a high level in recent years and maintain steady growth. In countries such as the United States, charitable giving started earlier, and individual donors have become the largest source of charitable donations. According to Giving USA (2021), individual donations accounted for 68.7% of total charitable giving of 324.10 billion dollars. Bennett and Sargeant (2005) also stress the need for more research on individual donors.

With the development of charity, effectively motivating and guiding donation behavior through communication strategies has become a research hotspot in philanthropy in recent years (Han et al., 2017; Goenka and Van Osselaer, 2019; Paramita et al., 2020). Among all kinds of communication methods to charity promotion, public service advertising is considered one of the most common and effective ways to stimulate the audience’s emotional response (Andreasen, 1993; Bagozzi and Moore, 1994). Children are one of the most frequently used characters in various types of donation appeals, such as charity advertisements with the themes of animal protection, poverty alleviation, disaster relief, educational projects, and medical assistance. For example, the Chinese Red Cross Foundation, one of the well-known charities in China, uses images of children as posters for the medical aid section of its website.^1^ The use of children’s images in fundraising is beneficial to enhance the effect of fundraising and raise more funds. Mingjuan Su’s poster^2^ with the slogan “I want to go to school” sparked a massive wave of support for the Hope Project, which has changed the lives of millions of children from low-income families in China. This photo moved millions of people and effectively expanded the awareness and influence of the Hope Project. By 2020, Hope Project has raised more than 17.58 billion yuan, supported more than 6.40 million needy students, and built more than 20,593 Hope Primary Schools, becoming one of China’s most widely engaged and influential public welfare projects. Why is the communication effect of children’s images exceptionally infectious and penetrating in donation appeals? What is the underlying mechanism for children’s images encouraging charitable donation intention? Is there any boundary condition for using images of children in donation appeals?

Children have more infant schema characteristics than adults in terms of physiological characteristics. According to the Stereotype Content Model (SCM, Fiske et al., 1999), these characteristics will affect people’s perception of the target and influence their attitude and behavior. This study focuses on the effect of infant schema cues, a specific charity promotion strategy, on donation intention. According to the theoretical model of empathic helping (Eisenberg et al., 2001), we will examine whether infant schema cues can enhance individual empathy and thus enhance their willingness to donate to charity. What is more, females have a better ability to capture infant schema cues and a higher level of empathy, which drives them to make more charitable donations than males. Based on that, we will also examine whether gender can moderate the positive effect of infant schema cues on charitable donation intention.

## Research Hypotheses

### Infant Schema Cues and Donation Intention

Infant schema, also known as baby schema and kindchenschema, refers to a specific set of physiological characteristics of infants, such as a large head, high forehead, round face, big eyes, small nose, small mouth, stubby limbs, and plump body shape (Lucion et al., 2017; Long et al., 2021). These characteristics are considered as cute and can generate people’s affection for infants, which motivates their caring and lead to caretaking behavior (Zebrowitz et al., 1993; Parsons et al., 2010). By using anthropometric data to compose different infants’ images, Glocker et al. (2009b) indicate that people perceive infants with more obvious infant schema characteristics (i.e., bigger eyes, round face, smaller mouth, and larger head) to be cuter. Sprengelmeyer et al. (2009) manipulate facial features in different positions and sizes. They show that people have different sensitivities to the perceived cuteness of infants when they are in varying levels of infant schema cues.

Adults have an attentional capture effect on infant schema features. They can distinguish infant and adult faces within 1 s, indicating that adults can quickly capture and process infant face features (Brosch et al., 2007). People can rapidly process the face features of infant schema, and such rapid processing is automated (McCall and Kennedy, 1980; Endendijk et al., 2018). From an evolutionary point of view, the infant schema features now considered more attractive may be formed by the continuation and development of good genes in evolution. Infant schema arouses adults’ attentional preference and leads to caretaking behaviors (Zebrowitz et al., 1993; Parsons et al., 2010). It can help the infant who cannot survive on his/her own get carefully nurtured, which is of great significance to the survival of the entire population (Long et al., 2021). Infant schema makes babies look more loveable, while loveable infants are considered friendly, healthy, and lovely. People are more likely to have caring and nurturing motivation toward them, which is not limited by kinship (Glocker et al., 2009a; Cheng et al., 2015).

The infant schema effect does not only exist in infants. The babyface generalization effect occurs when the image of a child or even an adult contains infant schema cues, which indicates that people react to children and adults with infant schema cues in a similar way to infants (Luo et al., 2011). For example, Bressan et al. (2009) mix adult and infant faces in various proportions to produce composite images. When the composite adult faces contain more cues of infantile characteristics, people rate them as more attractive. However, when the composite faces include more cues of adult characteristics, people rate them as less attractive.

From what has been discussed above, infant schema cues can lead to cuteness perception, attract people’s attention, and encourage prosocial behavior (Baeken et al., 2010). Prosocial behaviors refer to helping others, charitable giving, and self-sacrifice activities that have little chance of being rewarded (Aronson et al., 2010). From the perspective of psychological mechanisms, people tend to believe in the causal cycle and thus hold a positive attitude toward prosocial behaviors (Kulow and Kramer, 2016). As a typical representative of prosocial behavior, donation has been widely discussed in social psychology research. Donations refer to the acts of giving (money, time, or other resources) to non-interest-related individuals or organizations, and usually for charitable purposes (Bekkers and Wiepking, 2011). Charity donation is concerned with helping others, so people are more willing to donate when they are in a strong desire to help others (Kandaurova and Lee, 2019).

Accordingly, infant schema cues can drive people to take more caring and helping behaviors, thus improving their willingness to donate. Hence, we propose the hypothesis as follows:

*H1*: Compared with adult schema cues, infant schema cues can improve people’s donation intention.

### The Mediating Role of Empathy

We propose that empathy plays an essential role in the relationship between infant schema cues and donation intention. Cuteness perception triggered by infant schema cues could improve empathy (Steinnes et al., 2019), referring to the emotional experience of compassion and care for others in need (Dovidio et al., 2017). Infant schema cues can shorten the psychological distance between people and the object, which improves people’s need for belongingness and makes them have a strong emotional resonance and empathy (Lehmann et al., 2013). Driven by empathy, people have stronger prosocial motivation and higher intention for prosocial behavior (Baumeister et al., 2007).

Prior research documents that empathy is an essential factor in determining willingness to donate (Baumeister et al., 2007). Eisenberg et al. (2001) suggest that helping behavior begins with empathy for others’ pain, which is mediated by emotional resonance. That is to say, empathy is a vital prerequisite for helping recipients with difficulties. Thus, they propose a behavior model of “empathy → sympathy → helping behavior.” Empathy is not only a pity emotion to feel sympathetic for others but also a tendency to improve their well-being. Empathy for recipients can significantly increase people’s willingness to donate.

Empathy can be activated in many ways (Kandaurova and Lee, 2019). As a result, charity organizers can use effective appeals to inspire donors’ empathy. Bekkers and Wiepking (2011) identify persuasion as one of the fundamental elements determining donation behaviors. Since the significance of persuasion in encouraging people’s donation intention, charity institutions use different forms of charity appeals to persuade people to donate. Specifically, charity appeal refers to the fundraising information displayed by the charity institutions, which influences the donors’ donation intention by using specific images, texts, and information frames in the public service advertising (Bendapudi et al., 1996). For example, when the recipient’s image is displayed in a charity advertisement, the donor will be encouraged to empathize with the recipient and increase the number of individual donations (Hung and Wyer, 2009). In the above discussion of this study, we mentioned that infant schema cues could promote empathy. Therefore, we argue that adding infant schema cues to charity promotion can help increase people’s empathy and thus improve their willingness to donate.

Accordingly, we propose the following hypothesis:

*H2*: Empathy plays a mediating role in the positive relation between infant schema cues and donation intention.

### The Moderating Role of Gender

In addition, the infant schema effect is more significant in females when compared with males. The reasons can be understood from the perspective of evolution and the social division of labor (Lehmann et al., 2013). In the evolution of human society, females have undertaken more parenting responsibilities. Especially in the early parenting process, the difference is more pronounced. To ensure that females allocate adequate care and nurturing resources to young children, females have a more vital ability to recognize and capture infant schema cues and a higher level of empathy than males (Lobmaier et al., 2010).

The attention and recognition of infant schema cues are affected by differences in hormone secretion (Holtfrerich et al., 2018). Specifically, females have more neuropeptide oxytocin than males, which positively affects the activation of inferior frontal junctions. Such an activation enhances the selective attention to infant schema cues. While males have more testosterone than females, the increase in testosterone will reduce the capture and concentration of infant schema cues. In other words, females produce more hormones that capture infant schema cues than males. In addition, infant schema cues can activate the ventral tegmental area (VTA), putamen, anterior cingulate cortex (ACC), and other brain regions and positively affect caring motivation. This mechanism is enhanced after the increase of the level of oxytocin in the subjects (Bos et al., 2018). Thus, gender could influence the impact of empathy on individual donation intention, which is more significant among females (Kemp et al., 2013).

Accordingly, the above reasoning leads to the hypothesis:

*H3*: Gender plays a moderating role in the positive relation between infant schema cues and donation intention. Compared with males, infant schema cues have a more significant positive impact on females’ willingness to donate.

## Materials and Methods

We conducted four experimental studies to test the hypotheses. In Study 1, we examined the main effect of infant schema cues on donation intention. We found that infant schema cues were more effective than adult schema cues for improving participants’ donation intention under a time donation context. In Study 2, we designed charity posters using infant schema cues, cute schema cues, and adult schema cues to test whether infant schema cues are more effective in charity promotion under a money donation context than cute schema cues. Study 3 replicated the result and further examined the mediating role of empathy. It showed that infant schema cues improved participants’ empathy, positively predicting donation intention. Study 4 examined the moderating role of gender and showed that infant schema cues positively affected donation intention for females but not for males.

### Study 1: The Main Effect of Infant Schema Cues

In this study, we designed charity posters using infant schema cues vs. adult schema cues to test whether infant schema cues are more effective in charity promotion under a time donation context.

#### Participants

A total of 71 participants (28 males, 43 females, Mean Age = 30.06 years, *SD* = 8.22) were recruited from an online recruitment platform in China^3^ in exchange for monetary incentives. They were randomly assigned to the infant schema cues and adult schema cues conditions.

#### Procedure

This study used a two-condition (cues: infant schema vs. adult schema) between-subjects design. The participants were exposed to a poster for public service advertising, along with an introduction to a charity event with a family reunion theme. In the infant schema cues condition, the characters for the poster were three children, while three adults were in the adult schema cues condition. Both sets of characters were photographed from the same three persons’ childhood and adulthood (See Supplementary Presentation for details). Other factors in the two posters were the same. Participants were then asked to answer questions measuring donation intention and other control variables.

#### Measures

We used one item adapted from Kulow and Kramer (2016) to measure participants’ donation intention (i.e., If you have a certain amount of spare time every month, are you willing to use part of your spare time to go to our charity group to help prepare and participate in charity activities?). Participants were asked to indicate their willingness to donate time (1 = extremely unwilling, 7 = extremely willing).

We measured participants’ emotions with four items (Watson et al., 1988). Participants were asked to indicate to what extent they feel happy/excited/angry/sad (where 1 = not at all, 5 = totally; for the two positive emotions: *α* = 0.781; for the two negative emotions: *α* = 0.797).

#### Results and Discussion

There was no significant difference in positive emotions between infant schema cues condition and adult schema cues condition (*M*_infant schema_ = 3.06, *SD* = 0.97 vs. *M*_adult schema_ = 3.00, *SD* = 1.14; *p* = 0.804), but there were significant differences in negative emotions (*M*_infant schema_ = 2.13, *SD* = 0.99 vs. *M*_adult schema_ = 2.77, *SD* = 1.33; *p* = 0.022).

**Figure 1:**
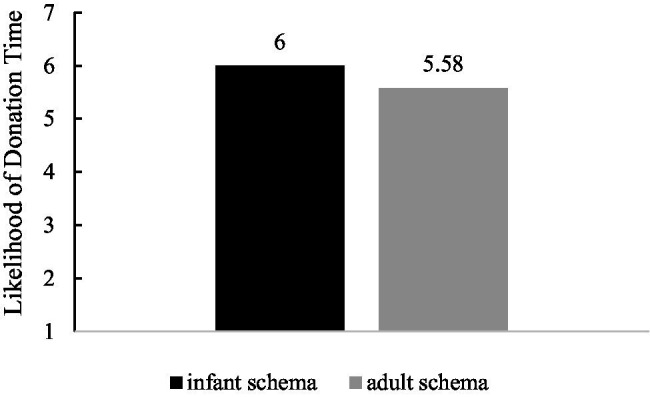
Influence of infant schema cues on donation intention.

We conducted a *t*-test analysis to examine the main effect of infant schema cues on donation intention. The results showed that there was a significant difference in donation intention between the two conditions [*t*(69) = 2.157, *p* = 0.034, *Cohen’s d* = 0.519]. Compared with the adult schema cues condition, the infant schema cues condition was more willing to donate time (*M*_infant schema_ = 6.00, *SD* = 0.75 vs. *M*_adult schema_ = 5.58, *SD* = 0.89). See Figure 1 for details.

The results of study 1 preliminarily showed that infant schema cues could lead to a higher donation intention compared with adult schema cues. However, there were two issues that needed to be addressed in this study. First, the children in this study had more perceived cuteness than the adults in the adult schema cues condition. Previous research has found that the perception of cuteness leads to more positive emotions and behaviors. Chang et al. (2017) and Wang et al. (2017) suggest that the cute design and image can enhance consumers’ sense of social responsibility and increase their willingness to consume green products. Langlois et al. (1995) find that adults are more affectionate with infants who have cuteness characteristics. We should further prove that the mere exposure to infant schema cues makes people have a higher donation intention not driven by cute cues. Second, the infant and adult schema cues significantly differed in age. Thus, we should rule out the alternative explanation of the impact of perceived age in posters on donation intention. In Study 2, we added cute schema cues condition without infant features to exclude perceived cuteness as an alternative explanation.

According to Kandaurova and Lee (2019), a monetary donation was different from a time donation because it had direct financial implications. Compared with time-related context, money-related context may activate a different cognitive mindset and thus influence people’s donation behaviors in a different way (Liu and Aaker, 2008). Existing literature has pointed out that when faced with money-related priming cues, people’s decisions and behaviors may differ from those encountered with unrelated tasks (Vohs et al., 2006). For example, Vohs et al. (2006) proposed that simple money-related priming cues could stimulate individuals to generate higher egoistic motivation and reduce individual prosocial behaviors. Jiang et al. (2014) also found similar results in their study, which showed that people were less likely to express their feelings in public after being reminded of money. However, some research indicated that money-related donation was also essential for donation behavior. Therefore, we further explored that infant schema cues could still improve donation intention under a situation of monetary donation in the following study.

### Study 2: Rule Out the Cute Schema Cues as Alternative Explanation

In this study, we designed charity posters using infant schema cues, cute schema cues, and adult schema cues to test whether infant schema cues are more effective in charity promotion under a money donation context rather than cute schema cues. In Study 2, we wanted to rule out the perceived cuteness of character in posters as an alternative explanation in the effect of infant schema cues on donation intention in charity promotion. We also aimed to show that the main effect of infant schema cues on donation intention would also exist in the monetary donation context.

#### Participants

A total of 146 participants (70 males, 76 females, Mean Age = 27.81 years, *SD* = 6.65) were recruited from an online recruitment platform in China^4^ in exchange for monetary incentives. They were randomly assigned to the infant schema cues, cute schema cues and adult schema cues conditions.

#### Procedure

Study 2 employed a three-condition (cues: infant schema vs. cute schema vs. adult schema) between-subjects design. The participants were exposed to a poster for public service advertising, along with an introduction to a charity event with a book donation theme. In the infant schema cues condition, the character for the poster was a child with a book, while a cute cartoon bear was in the cute schema cues condition. In the adult schema cues condition, there was an adult with a book in the poster. Both sets of characters in infant schema cues condition and in adult schema cues condition were photographed from the same person’ childhood and adulthood (See Supplementary Presentation for details). Other factors in the three posters were the same (See Supplementary Presentation for details). Then, participants were asked to answer questions measuring donation intention and other control variables.

#### Measures

We used one item adapted from Kulow and Kramer (2016) to measure participants’ donation intention (i.e., Assuming that you have already worked and your income is stable. If you have some spare money every month, how much would you be willing to donate for our public welfare activities and donate books?). Participants were asked to indicate how much they were willing to donate their money.

We also asked the participants to rate the perceived cuteness (1 = not cute at all, 7 = very cute) and the perceived age of the character in posters (1 = is very young, 7 = is very old). Finally, the participants also answered some demographic questions, such as gender and age.

#### Results and Discussion

We wanted to exclude the cuteness as an alternative explanation of the infant schema cues effect. We asked participants to rate the perceived cuteness of character in poster they have seen. Regressing the perceived cuteness on schema cues yielded a significant main effect [*F*(2,143) = 26.75, *p* < 0.001, *η*^2^ = 0.272]. Participants thought the infant schema cues were cuter than the adult schema cues [*M*_infant schema_ = 5.15, *SD* = 0.20; *M*_adult schema_ = 3.48, *SD* = 0.20; *F*(1,143) = 35.21, *p* < 0.001, *η*^2^ = 0.198]. While compared with the cute schema cues condition, there was no difference in perceived cuteness between infant schema cues and cute schema cues [*M*_infant schema_ = 5.15, *SD* = 0.20; *M*_cute schema_ = 5.31, *SD* = 0.19; *F*(1,143) = 0.32, *p* = 0.575, *η*^2^ = 0.002]. In addition, participants also thought the cute schema cues were cuter than the adult schema cues [*M*_cute schema_ = 5.31, *SD* = 0.19; *M*_adult schema_ = 3.48, *SD* = 0.20; *F*(1,143) = 44.70, *p* < 0.001, *η*^2^ = 0.238].

**Figure 2:**
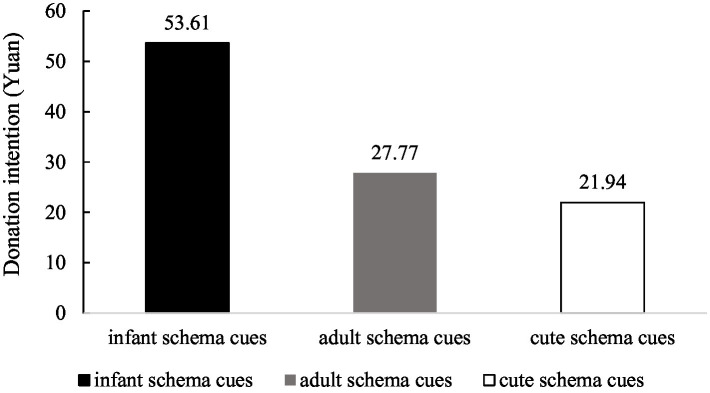
Influence of infant schema cues on donation intention in Study 2.

Even though there was no difference in the perceived cuteness between infant schema cues and cute schema cues, participants also had a higher donation intention in the infant schema cues condition than participants in the cute schema cues condition. We conducted an ANOVA analysis with donation intention as the dependent variable and schema cues as the independent variable. Regressing the donation intention on schema cues yielded a significant main effect [*F*(2,143) = 8.94, *p* < 0.001, *η*^2^ = 0.111]. Supporting our hypothesis, compared with the adult schema cues condition, participants in the infant schema cues condition were willing to donate more money [*M*_infant schema_ = 53.61, *SD* = 5.74; *M*_adult schema_ = 27.77, *SD* = 5.62; *F*(1,143) = 10.35, *p* = 0.002, *η*^2^ = 0.067]. While compared with the cute schema cues condition, participants in the infant schema cues condition were willing to donate more money [*M*_infant schema_ = 53.61, *SD* = 5.74; *M*_cute schema_ = 21.94, *SD* = 5.40; *F*(1,143) =16.15, *p* < 0.001, *η*^2^ = 0.101]. However, the willingness to donate had no difference between the cute schema cues condition and the adult schema cues condition [*M*_cute schema_ = 21.94, *SD* = 5.40; *M*_adult schema_ = 27.77, *SD* = 5.62; *F*(1,143) = 0.56, *p* = 0.456, *η*^2^ = 0.004]. See Figure 2 for details.

Participants needed to evaluate the perceived age of character in posters. After controlling the perceived age the participants rated, the effect of the donation intention on schema cues still yielded a significant main effect [*F*(2,142) = 9.34, *p* < 0.001, *η*^2^ = 0.116]. Supporting our hypothesis, compared with the adult schema cues condition, participants in the infant schema cues condition were willing to donate more money [*M*_infant schema_ = 54.72, *SD* = 5.86; *M*_adult schema_ = 25.13, *SD* = 6.28; *F*(1,142) = 10.88, *p* = 0.001, *η*^2^ = 0.071]. While compared with the cute schema cues condition, participants in the infant schema cues condition were willing to donate more money [*M*_infant schema_ = 54.72, *SD* = 5.86; *M*_cute schema_ = 23.40, *SD* = 5.62; *F*(1,142) =15.75, *p* < 0.001, *η*^2^ = 0.100]. However, the willingness to donate had no difference between the cute schema cues condition and the adult schema cues condition [*M*_cute schema_ = 23.40, *SD* = 5.62; *M*_adult schema_ = 25.13, *SD* = 6.28; *F*(1,142) = 0.04, *p* = 0.846, *η*^2^ < 0.001].

The results of study 2 showed that infant schema cues could lead to a higher donation intention than cute schema cues and adult schema cues. This effect persisted after controlling for the perceived age of the character in posters. However, there were two issues that needed to be addressed in this study. First, participants in the infant schema cues condition and the adult schema cues condition significantly differed in negative emotions. Thus, we should rule out the alternative explanation that emotion mediated the impact of infant schema cues on donation intention. Second, the children and adults in this study were presented as fundraising objects. We should prove further that only the presence of infant schema cues could inspire people to have a higher donation intention even when other objects were used as fundraising objects. Therefore, in Study 3, we changed the experimental stimulus and separated the fundraising object (i.e., stray dogs) of the charity activity from the character (i.e., children or adults) to explore that the mere exposure to infant schema cues in the poster could lead to a higher donation intention. In addition, Study 3 aimed to explore the mediating role of empathy.

### Study 3: The Mediating Role of Empathy

Study 3 aimed to address the issues raised in Study 2 and examined the mediating role of empathy in the relation between infant schema cues and donation intention. As we proposed, even if the infant schema was not the main character of the public service advertising, mere exposure to infant schema cues could stimulate people to have a higher donation intention. Therefore, in order to examine the robustness and generalizability of our main effect, we changed the experimental stimulus and no longer used children or adults as the main target of charity activities.

#### Participants

A total of 116 participants (55 males, 61 females, Mean Age = 31.78, *SD* = 9.42) were recruited from the online recruitment platform in China in exchange for monetary incentives. The participants were randomly assigned to the infant schema cues and adult schema cues conditions.

#### Procedure

This study used a two-condition (cues: infant schema vs. adult schema) between-subjects design. The participants were exposed to a poster for public service advertising, along with an introduction to a charity event with a stray-dogs caring theme. Both posters portrayed and fundraised for a stray dog. In the infant schema cues condition, the dog was led by a child, while an adult woman led the dog in the adult schema condition. Both characters in the two posters were photographed from the same person’s childhood and adulthood. Other factors in the two posters were the same. Participants were then asked to answer questions measuring donation intention and other control variables.

#### Measures

We used one item adapted from Kulow and Kramer (2016) to measure participants’ donation intention (i.e., If you have a certain amount of spare time every month, are you willing to use part of your spare time to participate in our public welfare activities at the stray animal rescue center?). Participants were asked to indicate their willingness to donate time (where 1 = extremely unwilling, 7 = extremely willing).

The measurement of empathy was adapted from the empathy subscale in the interpersonal response scale (Davis, 1983). Two of the items were selected and appropriately adapted according to the donation situation required by this study: (1) I can empathize with the suffering of those unfortunate animals; (2) I feel a strong bond with these unfortunate animals. Participants were asked to indicate to what extent they agree with the two statements (where 1 = strongly disagree, 7 = strongly agree; *α* = 0.719).

We measured participants’ emotions with four items (Watson et al., 1988), just the same as in Study 1. Participants were asked to indicate to what extent they feel happy/excited/angry/sad (where 1 = not at all, 5 = very; for the two positive emotions: *α* = 0.816; for the two negative emotions: *α* = 0.684).

#### Results and Discussion

We conducted an ANOVA analysis with donation intention as the dependent variable and infant schema cues as the independent variable. The results of the ANOVA yielded a significant effect of infant schema cues on donation intention [*F*(1,114) = 4.058, *p* = 0.046, *η*^2^ = 0.034]. Specifically, compared with the adult schema cues condition, participants in the infant schema cues condition were more willing to donate their time (*M*_infant schema_ = 5.75, *SD* = 1.14; *M*_adult schema_ = 5.30, *SD* = 1.25).

In order to rule out the possible influence of emotion on the effect, we also examined the main effect of infant schema cues on emotion. An ANOVA test showed that there was no significant difference in positive emotion between the infant schema condition and adult schema condition [*M*_infant schema_ = 2.70, *SD* = 1.04 vs. *M*_adult schema_ = 2.77, *SD* = 1.01; *F*(1,114) = 0.130, *p* = 0.719] and no significant difference in negative emotions [*M*_infant schema_ = 2.00, *SD* = 0.99 vs. *M*_adult schema_ = 2.10, *SD* = 1.04; *F*(1,114) = 0.263, *p* = 0.609]. No mediating effect was found of emotions in the influence of infant schema cues on donation intention.

**Figure 3:**
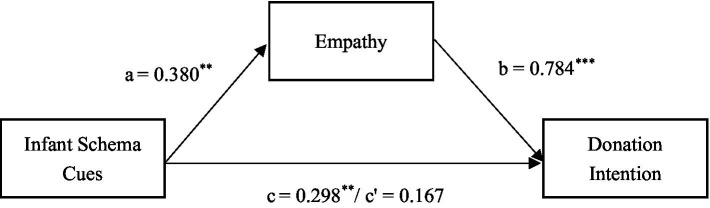
Mediating test of the influence of infant schema on donation intention. ^***^p < 0.001, ^**^p < 0.05; c represents the coefficient of direct effect, c’ represents the coefficient of indirect effect.

We used the PROCESS method (Hayes, 2017) to examine the mediating role of empathy in the effect of infant schema cues on donation intention. Model 4 was conducted with a repeated sample number of 5,000 and a CI of 95%. We took the infant schema cues as the independent variable, empathy as the mediating variable, donation intention as the dependent variable, and emotions as the control variable. The results showed that infant schema cues significantly and positively predicted empathy (*β* = 0.380, *t* = 2.158, *p* = 0.033, *CI* = [0.0310, 0.7281]). There was a significant positive effect of empathy on donation intention (*β* = 0.784, *t* = 8.310, *p* < 0.001, *CI* = [0.5973, 0.9714]), and the direct effect of infant schema cues on donation intention was no longer significant. The indirect effect of empathy on donation intention did not include 0 (*β* = 0.298, *CI* = [0.0329, 0.6080]). Empathy played an entirely mediating role in the influence of infant schema cues on donation intention (See Table 1 and Figure 3 for details).

**Table 1:** Model coefficients for the mediation model (empathy) in Study 3.

		Consequent						
		*M* (empathy)				*Y* (donation intention)		
Antecedent		Coeff.	*SE*	*p*		Coeff.	*SE*	*p*
*X* (infant schema cues)	*a*	0.38	0.18	0.033^**^	*c’*	0.17	0.18	0.355
*M* (empathy)		-	-	-	*b*	0.78	0.09	<0.001^***^
Covariate (positive emotion)		0.08	0.09	0.384		0.04	0.09	0.610
Covariate (negative emotion)		0.22	0.09	0.013^**^		−0.07	0.09	0.418
Constant	*i_M_*	4.41	0.33	<0.001^***^	*i_Y_*	1.34	0.53	0.013^**^
		*R*^2^ = 0.09 *F*(3,112) = 3.75 *p* = 0.013^**^				*R*^2^ = 0.41 *F*(4,111) = 19.59 *p* < 0.001^***^		

X: 1 = infant schema cues, 0 = adult schema cues.

*** *p* < 0.001;

** *p* < 0.05.

Study 3 showed that infant schema cues led people to higher empathy levels and thus increased their donation intention for charity promotion under the time donation context. To ensure the robustness and generalizability of the effect, we excluded the alternative explanation of emotion as a mediator in this study and no longer took infant and adult schema cues as the main character of the charity promotion.

As we proposed, the physiological characteristics of infant schema cues could affect people’s perception of the target object, enhance empathy, and then improve donation intention. Such a process was not restricted to donating time or money. In the subsequent study, we aimed to explore the moderating role of gender in the relationship between infant schema cues and donation intention.

### Study 4: The Moderating Effect of Gender

We had three primary purposes for Study 4. First, our results so far indicated that infant schema cues could motivate people to have a higher empathy under the context of time donation and monetary donation, thus enhancing people’s donation intention for related and unrelated objects. Second, although the infant schema cues or the adult schema cues were not infused into the fundraising objects of the charity publicity event, the character with these cues still played an essential role in the posters of public service advertising. Thus, we separated the infant schema cues from the charity events in Study 4 to show that mere exposure to infant schema cues could increase people’s donation intention, even if it was not directly related to the charity activity. Last, Study 4 also aimed to explore the moderating role of gender in the relationship between infant schema cues and donation intention. Behavioral and neural evidence shows sex differences when people respond to infant schema. Glocker et al. (2009a) and Lehmann et al. (2013) find that although both females and males find infant schema cute, females are more sensitive to the cuteness of infant schema and show a higher preference for them compared to males (Charles et al., 2013). According to Schwartz (1992), females have more benevolent than males, which is an indicator to measure people’s social orientation of caring for the well-being of others. So, when exposed to infant schema cues, females may act differently from males. To better understand whether gender plays a moderating role of gender in the relationship between infant schema cues and donation intention, we design Study 4.

#### Participants

A total of 136 participants (71 males, 65 females, Mean Age = 29.15, *SD* = 6.88) were recruited from the online recruitment platform in China in exchange for monetary incentives. They were randomly assigned to infant and adult schema cues conditions.

#### Procedure

We divided this study into two parts. We manipulated the image cues (infant schema vs. adult schema) in the first part. Participants were shown an image photographed in a person’s childhood or adulthood according to the conditions (See Supplementary Presentation for details). Other elements in the two images were kept the same. We disguised this process as an attention check task, in which participants were asked to point out what the image depicted. This step aimed to prime participants’ perceptions of infant or adult schema cues.

In the second part, participants would see an introduction with a poster for a charity activity, which appealed to caring for the empty-nest elderly initiated by Beijing Great Love Foundation (a fictitious organization). They then answered their donation intentions.

#### Measures

We used one item adapted from Kulow and Kramer (2016) to measure participants’ donation intention (i.e., Assuming that you have already worked and your income is stable. If you have some spare money every month, are you willing to donate some of your spare money to join our public welfare activities and help these lonely older people?). Participants were asked to indicate how much they were willing to donate their money. Finally, participants were asked to answer some demographic questions.

#### Results and Discussion

A two-way ANOVA, with donation amount as the dependent variable, and schema cues and gender as independent variables, yielded main effect for schema cues [*F*(1,132) = 4.597, *p* = 0.034, *η*^2^ = 0.034] but no main effect for gender [*F*(1,132) = 0.722, *p* = 0.397]. Notably, the interaction of schema cues and gender was significant [*F*(1,132) = 5.835, *p* = 0.017, *η*^2^ = 0.042].

**Figure 4:**
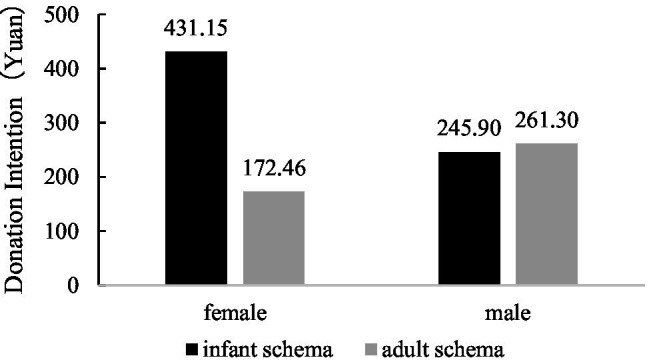
Influence of infant schema cues on female and male donation intention.

Simple effects were further analyzed. The main effect of schema cues was significant for female participants [*F*(1,132) = 9.878, *p* = 0.002, *η*^2^ = 0.070]. Specifically, infant schema cues led to a higher amount of money the female participants were willing to donate (*M*_infant schema_ = 431.15, *SD* = 63.76 vs. *M*_adult schema_ = 172.46, *SD* = 52.06). However, the main effect of schema cues was not significant for male participants [*F*(1,132) = 0.039, *p* = 0.844]. There was no significant difference between the infant schema condition and the adult schema condition for male participants (*M*_infant schema_ = 245.90, *SD* = 50.77 vs. *M*_adult schema_ = 261.30, *SD* = 59.35). See Figure 4 for details.

This study showed that the main effect of infant schema cues on donation intention would also exist in the context of monetary donation. Even if the infant schema cues were separated from the charity donation event, mere exposure to the infant schema cues could improve people’s donation intention. This study also found that the effect of infant schema cues on donation intention was more robust for females than for males.

## Discussion and Limitation

This paper examined the effect of infant schema cues on the donation intention in charity promotion. We performed four experiments and found that due to a babyface overgeneralization effect, the infant schema cues could inspire people to have a higher level of empathy, while activating empathy would enhance people’s prosocial motives and improve their donation intention. The effect of infant schema cues on donation intention appeared both in time donation and monetary donation. We also found that cuteness did not play a role in this effect. What is more, females were more likely to capture infant schema cues and prime a higher level of empathy than males. Hence, we found that the main effect of infant schema cues on donation intention was more significant in females than in males. This paper found that infant schema cues could not only improve people’s donation intention in related situations and objects but also in unrelated situational tasks.

This research makes several theoretical contributions. First, we enrich the literature on infant schema research. Previous literature on infant schema mainly focuses on the influence of infant schema features of human beings, animals, and other living beings on people’s psychological cognition (Berry and McArthur, 1985; Long et al., 2021), emotion responses (Almanza-Sepdulveda et al., 2018), and evaluations (Proverbio, 2017), which indicates an attentional bias effect caused by infant schema on the characteristics of target objects (Brosch et al., 2007; Jia et al., 2017). Few studies discuss how infant schema cues influence people’s behavioral intentions. Furthermore, previous research studies how people react toward a specific baby or adult image (Glocker et al., 2009a; Lucion et al., 2017), but few extend the infant schema cues into a practical context. We infuse the infant schema cues into public service advertising designs, which could serve as an essential bridge between the research domains of infant schema and charitable donation.

Second, we also find that the mere presence of images with baby characteristics can elicit positive responses and increase people’s willingness to donate. Prior studies document that charity appeals have a significant persuasive effect. When the recipients appear as the protagonist in the charity appeal poster, it can arouse people’s closer psychological distance and emotional resonance, thus leading to a higher level of empathy and willingness to donate (Bendapudi et al., 1996; Hung and Wyer, 2009). Study 3 demonstrates that even though infant schema cues are used as peripheral cues in the charity appeals instead of core cues, people’s empathy and donation intention will still be enhanced. Study 4 finds that even mere exposure to the infant schema cues can also lead to a higher willingness to donate to unrelated charity activities. The findings in our paper enrich the research on the effects of charity appeals and persuasion.

Third, this study also contributes to the literature on prosocial behavior. Donation intention is one of the critical factors in prosocial behavior research (Kandaurova and Lee, 2019), and an increasing body of literature has studied how to effectively motivate and guide donation behavior through communication strategies (Han et al., 2017; Goenka and Van Osselaer, 2019; Paramita et al., 2020). This paper finds the positive impact of infant schema cues on donation intention. We also document the mediating role of empathy and the moderating role of gender, conducive to expanding the whole theory of prosocial behavior.

The conclusions offer critical practical implications for leveraging the influence of infant schema cues on donation intention in charity activities. When foundations and other charitable organizations shoot public service advertising and call on people to carry out prosocial behaviors, they can appropriately include the image of infants or children as the main body of the advertisement to get more donations. Similarly, the image of infants or children can also be used in crowdfunding. In addition to providing basic information (i.e., products, innovation, and company history), product promotion can also use infant schema cues to arouse the audience’s empathy to gain recognition and improve the possibility of success. What is more, we find that compared with males, infant schema cues have a more significant effect on enhancing females’ empathy and donation intention. Therefore, introducing infant schema cues may achieve better results when designing charity activities for female groups.

There are still some limitations suggesting further research. First, we investigate the effect of infant schema cues on donation intention through experimental scenarios rather than using a field study. Thus, future research can use a field setting in an actual donation situation to test the practical application of our effect and increase the validation of the results.

Second, in addition to gender, other individual characteristics may also influence the relation between infant schema cues and donation intention. For example, participants’ own marital and reproductive status may lead to different levels in their willingness to donate after capturing infant schema cues. Thus, individuals’ personality characteristics can also be considered in the future.

Finally, the effect of camera language (e.g., depth of field: close vs. far) on children’s images can be considered. In practical applications, infant schema is often accompanied by close-up shots, with children’s or infants’ faces taking up a larger proportion of the images, while other schema cues are often accompanied by distant shots. Therefore, whether there is a match between camera language and infant schema cues can be further studied.

## Data Availability Statement

The raw data supporting the conclusions of this article will be made available by the authors, without undue reservation.

## Author Contributions

CX, CY, and JL contributed to the conception and design of the study. MZ and JL wrote the protocol. CX and CY were responsible for data collection and conducted the statistical analyses, under the supervision of MZ. MZ, CX, and JL wrote the first draft of the manuscript. CY wrote the revised manuscript. All authors edited subsequent drafts and approved the submitted version. All authors contributed equally to this work.

## Funding

This research was funded by the Zhejiang A&F University Talent Initiative Project (W20220028). This funding supports experimental fees and open access publication fees. CX acknowledges the support from the China Scholarship Council (202006360159).

## Conflict of Interest

The authors declare that the research was conducted in the absence of any commercial or financial relationships that could be construed as a potential conflict of interest.

## Publisher’s Note

All claims expressed in this article are solely those of the authors and do not necessarily represent those of their affiliated organizations, or those of the publisher, the editors and the reviewers. Any product that may be evaluated in this article, or claim that may be made by its manufacturer, is not guaranteed or endorsed by the publisher.

## Footnotes

^1^ https://www.crcf.org.cn/

^2^In the black and white photo, the 8-year-old girl, shabbily dressed and with disheveled hair, sits at a table with a pencil in her hand and a timid expression on her face. A pair of pure eyes seem to be staring at you, revealing an eager light.

^3^ https://www.wjx.cn/

^4^ https://www.credamo.com/
