# Supplementary material for: The Influence of Infant Schema Cues on Donation Intention in Charity Promotion
Source: Front Psychol. 2022 Jul 12;13:869458. doi: 10.3389/fpsyg.2022.869458 (PMC9326485; doi:10.3389/fpsyg.2022.869458)
Supplement: Supplementary file 1 [file Presentation_1.pdf]

The influence of infant schema cues on donation intention in charity promotion

Chen Yang[†], Mengying Zhao[†], Chunya Xie[†] and Jingyi Li[*†]

This supplementary Web Appendix includes detailed figures on the experiments reported throughout the paper. It describes the exact stimuli used in four studies we reported in the paper. We took photographer Irina Werning's project, *Back to The Future* (readers can see this wonderful project in *https://irinawerning.com/back-to-the-future/*), as our posters' characters and added cover stories of these images as our stimuli. If readers are interested in more detail, or copies of the manipulations or other study materials, please contact the corresponding author, Jingyi Li (ruclijingyi@foxmail.com).

Study 1: The main effect of infant schema cues

The posters which all participants saw were about family reunions. In the infant schema cues condition, the characters for the poster were three children, while three adults were in the adult schema cues condition. Both sets of characters were photographed from the same three persons' childhood and adulthood. Other factors in the two posters were the same (see Figure 1 for details).

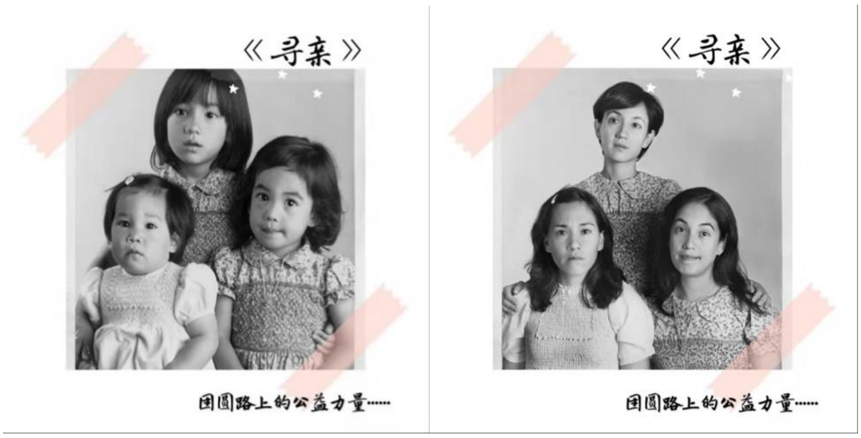

Source: [Irina Werning's project, *Back to The Future*, https://irinawerning.com/back-to-the-future/]. Reproduced with permission.

Note. "寻亲" means "Find relatives"; "团圆路上的公益力量" means "Public welfare power in family reunion"

Figure 1. The posters for a family-reunion-themed charity event in Study 1

(infant schema cues vs. adult schema cues)

Study 2: **Rule out the cute schema cues as alternative explanation**

The posters which all participants saw were about book donation. In the infant schema cues condition, the character for the poster was a child with a book, while a cute cartoon bear was in the cute schema cues condition. In the adult schema cues condition, there was an adult with a book in the poster. Other factors in the three posters were the same (see Figure 2 for details).

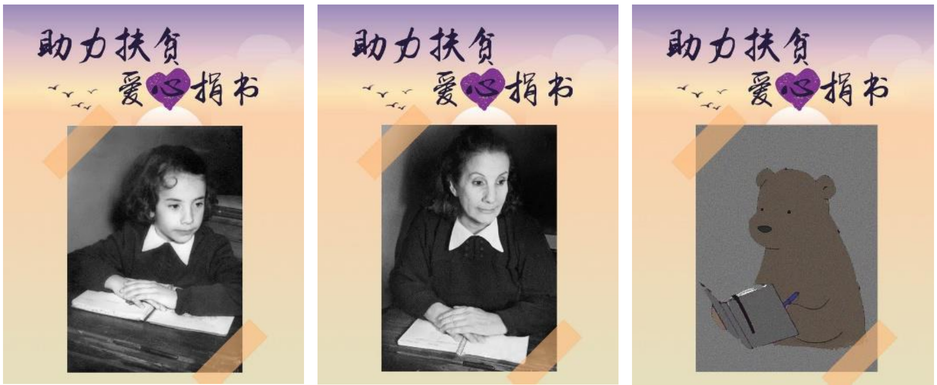

Source: [Irina Werning’s project, *Back to The Future*, https://irinawerning.com/back-to-the-future/]. Reproduced with permission.

Note. “助力扶贫” means “helping poverty alleviation”; “爱心捐书” means “donating books”

Figure 2. The posters for a book-donation charity event in Study 2

(infant schema cues vs. adult schema cues vs. cute schema cues)

Study 3: **The mediating role of empathy**

The posters which all participants saw were about stray-dogs caring. In the infant schema cues condition, the dog was led by a child, while an adult woman led the dog in the adult schema condition. Both characters in the two posters were photographed from the same person’s childhood and adulthood. Other factors in the two posters were the same (see Figure 3 for details).

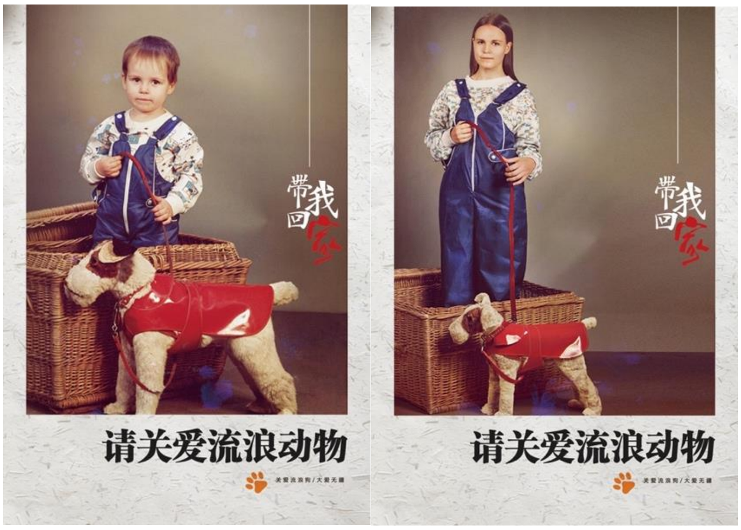

Source: [Irina Werning's project, *Back to The Future*, https://irinawerning.com/back-to-the-future/]. Reproduced with permission.

Note. "带我回家" means "Take me home"; "请关爱流浪动物" means "Please care for stray animals"

Figure 3. The posters for a stray-dogs-caring themed charity event in Study 3

(infant schema cues vs. adult schema cues)

Study 4: The moderating effect of gender

Participants were shown an image photographed in a person's childhood or adulthood according to the conditions. Other elements in the two images were kept the same (see Figure 4 for details).

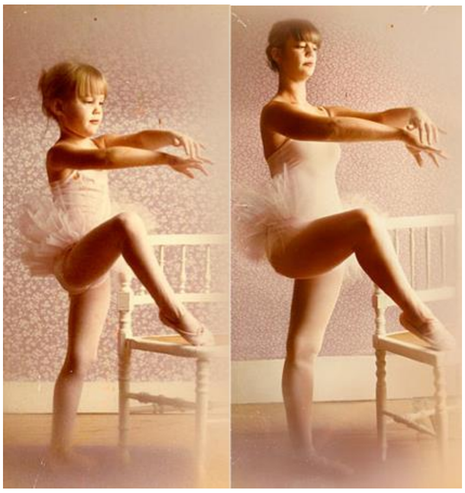

Source: [Irina Werning's project, *Back to The Future*, https://irinawerning.com/back-to-the-future/]. Reproduced with permission.

Figure 4. The stimulus for Study 4 (infant schema cues vs. adult schema cues)
